# Supplementary material for: Constraining neutron-star matter with microscopic and macroscopic collisions
Source: Nature. 2022 Jun 8;606(7913):276–80. doi: 10.1038/s41586-022-04750-w (PMC9177417; doi:10.1038/s41586-022-04750-w)
Supplement: Supplementary file 1 — This file contains legends for Supplementary Tables 1–5. [file 41586_2022_4750_MOESM1_ESM.pdf]

nature portfolio

https://doi.org/10.1038/s41586-022-04750-w

Supplementary information

Constraining neutron-star matter with microscopic and macroscopic collisions

In the format provided by the authors and unedited

Supplementary Table 1 | Kullback–Leibler divergence with different observation input.

$D_{\rm KL}$	Astro only	HIC only	Astro+HIC
$1.0n_{\rm sat}$	0.079	0.109	0.270
$1.5n_{\rm sat}$	0.075	0.108	0.266
$2.0n_{\rm sat}$	0.112	0.019	0.174
$2.5n_{\rm sat}$	0.244	0.006	0.274
$1.0M_\odot$	0.090	0.054	0.128
$1.4M_\odot$	0.185	0.022	0.210
$1.6M_\odot$	0.225	0.015	0.251
$2.0M_\odot$	0.228	0.008	0.222

[1] Comparison of the Kullback–Leibler divergence (KL divergence) $D_{\rm KL}(\text{posterior}|\text{prior})$ of pressure and radius of a neutron star with respect to the prior in bits when including only astrophysical constraints, only HIC experimental data, and for the combination of both. The KL divergence quantifies the additional information encoded in the posterior distribution with respect to the prior distribution. A KL divergence of zero indicates that the two distributions are identical. For reference, the KL divergence $D_{\rm KL}(\mathcal{N}(0,1/4)|\mathcal{N}(0,1)) \approx 1.2\text{bits}$, where $N(\mu,\sigma^2)$ is a normal distribution with mean μ and variance σ^2. The KL divergence for the pressure at $1.0n_{\rm sat}$ and $1.5n_{\rm sat}$, using only HIC experimental input, is higher than that of the result using only astrophysical observations. Therefore, the HIC experiment has higher impact than astrophysical observations for pressures below $1.5n_{\rm sat}$.

Supplementary Table 2 | Impact of the parameterisation for symmetric nuclear matter.

	SNM form used here		Taylor expansion	
P/R	HIC only	Astro+HIC	HIC only	Astro+HIC
$1.0n_{\rm sat}$	$2.05^{+0.49}_{-0.45}$	$2.11^{+0.49}_{-0.52}$	$1.95^{+0.52}_{-0.44}$	$2.01^{+0.51}_{-0.47}$
$1.5n_{\rm sat}$	$6.06^{+1.85}_{-2.04}$	$6.25^{+1.90}_{-2.26}$	$5.61^{+2.04}_{-2.00}$	$5.87^{+1.99}_{-2.14}$
$2.0n_{\rm sat}$	$19.47^{+33.63}_{-11.67}$	$19.07^{+15.27}_{-10.53}$	$18.80^{+32.63}_{-12.89}$	$18.72^{+16.57}_{-9.34}$
$2.5n_{\rm sat}$	$47.78^{+75.96}_{-32.96}$	$45.43^{+40.41}_{-19.11}$	$47.58^{+77.40}_{-31.93}$	$45.66^{+41.66}_{-19.19}$
$1.0M_\odot$	$11.89^{+0.79}_{-0.98}$	$11.88^{+0.57}_{-0.76}$	$11.77^{+0.84}_{-0.97}$	$11.79^{+0.60}_{-0.71}$
$1.4M_\odot$	$12.06^{+1.13}_{-1.18}$	$12.01^{+0.78}_{-0.77}$	$11.98^{+1.16}_{-1.18}$	$11.97^{+0.77}_{-0.74}$
$1.6M_\odot$	$12.11^{+1.33}_{-1.33}$	$12.03^{+0.98}_{-0.75}$	$12.05^{+1.32}_{-1.37}$	$12.00^{+0.90}_{-0.78}$
$2.0M_\odot$	$12.19^{+1.71}_{-1.59}$	$11.91^{+1.24}_{-1.11}$	$12.13^{+1.73}_{-1.61}$	$11.92^{+1.23}_{-1.10}$

[2] Comparison of the 95% credible interval for the pressure $[\,\mathrm{MeV\,fm^{-3}}]$ and radius [km] of neutron stars when including only HIC experiments and for combined HIC and astrophysics results for two parameterisations of symmetric nuclear matter. In particular, we compare the functional form from FOPI used in this work, see Eq. (5), with a general Taylor expansion for symmetric nuclear matter with the same values for the saturation point and the incompressibility but including the third-order parameter $Q = -150 \pm 250\,\mathrm{MeV}$ at 1σ using a Gaussian distribution. We find that our results are robust with respect to a variation of this parameterisation and the impact of this choice is at the 5% level for pressures and 1% level for radii.

Supplementary Table 3 | Impact of the proton fraction in β-equilibrium.

	$x_{\rm ASY\text{-}EOS}$		$0 \leq x \leq 0.1$	
P/R	HIC only	Astro+HIC	HIC only	Astro+HIC
$1.0n_{\rm sat}$	$2.05^{+0.49}_{-0.45}$	$2.11^{+0.49}_{-0.52}$	$2.05^{+0.50}_{-0.45}$	$2.10^{+0.48}_{-0.52}$
$1.5n_{\rm sat}$	$6.06^{+1.85}_{-2.04}$	$6.25^{+1.90}_{-2.26}$	$6.02^{+1.89}_{-2.04}$	$6.23^{+1.81}_{-2.31}$
$2.0n_{\rm sat}$	$19.47^{+33.63}_{-11.67}$	$19.07^{+15.27}_{-10.53}$	$19.32^{+33.95}_{-11.05}$	$19.00^{+14.74}_{-10.54}$
$2.5n_{\rm sat}$	$47.78^{+75.96}_{-32.96}$	$45.43^{+40.41}_{-19.11}$	$48.00^{+78.57}_{-34.40}$	$45.48^{+39.96}_{-19.28}$
$1.0M_\odot$	$11.89^{+0.79}_{-0.98}$	$11.88^{+0.57}_{-0.76}$	$11.88^{+0.79}_{-0.98}$	$11.87^{+0.59}_{-0.75}$
$1.4M_\odot$	$12.06^{+1.13}_{-1.18}$	$12.01^{+0.78}_{-0.77}$	$12.05^{+1.14}_{-1.17}$	$12.00^{+0.77}_{-0.77}$
$1.6M_\odot$	$12.11^{+1.33}_{-1.33}$	$12.03^{+0.98}_{-0.75}$	$12.10^{+1.31}_{-1.36}$	$12.03^{+0.91}_{-0.79}$
$2.0M_\odot$	$12.19^{+1.71}_{-1.59}$	$11.91^{+1.24}_{-1.11}$	$12.18^{+1.70}_{-1.61}$	$11.90^{+1.22}_{-1.14}$

[3] Comparison of the 95% credible interval for the pressure $[\,\mathrm{MeV\,fm^{-3}}]$ and radius [km] of neutron stars when including only HIC experiments and for combined HIC and astrophysics results for two choices for the proton fraction in β-equilibrium. For the main results, we compute the proton fraction for the HIC constraints using the EOS functional introduced by the ASY-EOS analysis ($x_{\rm ASY\text{-}EOS}$). We compare this with a more conservative choice that constrains the proton fraction to be within the range $0 \leq x \leq 0.1$ but find only small changes.

Supplementary Table 4 | Impact of the radius constraints for J0740+6620.

		Using Ref.[7, 8] for J0740+6220		Using Ref.[38] for J0740+6220	
P/R	HIC only	Astro only	Astro+HIC	Astro only	Astro+HIC
$1.0n_{\rm sat}$	$2.05^{+0.49}_{-0.45}$	$2.00^{+0.52}_{-0.49}$	$2.11^{+0.49}_{-0.52}$	$1.95^{+0.55}_{-0.45}$	$2.08^{+0.49}_{-0.53}$
$1.5n_{\rm sat}$	$6.06^{+1.85}_{-2.04}$	$5.84^{1.96}_{-2.26}$	$6.25^{+1.90}_{-2.26}$	$5.63^{+2.16}_{-2.05}$	$6.14^{+1.93}_{-2.28}$
$2.0n_{\rm sat}$	$19.47^{+33.63}_{-11.67}$	$18.44^{+16.24}_{-9.69}$	$19.07^{+15.27}_{-10.53}$	$17.46^{+15.66}_{-9.27}$	$18.32^{+14.87}_{-9.60}$
$2.5n_{\rm sat}$	$47.78^{+75.96}_{-32.96}$	$45.05^{+39.80}_{-19.62}$	$45.43^{+40.41}_{-19.11}$	$42.23^{+41.75}_{-20.47}$	$43.22^{+42.66}_{-19.18}$
$1.0M_\odot$	$11.89^{+0.79}_{-0.98}$	$11.76^{+0.65}_{-0.71}$	$11.88^{+0.57}_{-0.76}$	$11.68^{+0.71}_{-0.74}$	$11.82^{+0.68}_{-0.78}$
$1.4M_\odot$	$12.06^{+1.13}_{-1.18}$	$11.94^{+0.79}_{-0.78}$	$12.01^{+0.78}_{-0.77}$	$11.83^{+0.86}_{-0.86}$	$11.94^{+0.87}_{-0.83}$
$1.6M_\odot$	$12.11^{+1.33}_{-1.33}$	$11.98^{+0.93}_{-0.79}$	$12.03^{+0.98}_{-0.75}$	$11.87^{+1.01}_{-0.93}$	$11.95^{+1.01}_{-0.91}$
$2.0M_\odot$	$12.19^{+1.71}_{-1.59}$	$11.88^{+1.23}_{-1.10}$	$11.91^{+1.24}_{-1.11}$	$11.74^{+1.44}_{-1.25}$	$11.77^{+1.42}_{-1.23}$

4 Comparison of the 95% credible interval for the pressure $[\,\mathrm{MeV\,fm^{-3}}]$ and radius [km] of neutron stars when including only HIC results, only astrophysical observations, and for combined HIC and astrophysics results when we include the combined mass-radius measurement from NICER [7, 8] or only the radio mass measurement from Ref. [38]. The radius of J0740+6620 estimated by NICER is preferring a stiffer EOS, which agrees well with the constraint from HIC experiments.

Supplementary Table 5 | Impact of excluding Danielewicz *et al.*[27].

	With Danielewicz *et al.*[27]		Without Danielewicz *et al.*[27]	
P/R	HIC only	Astro+HIC	HIC only	Astro+HIC
$1.0n_{\rm sat}$	$2.05^{+0.49}_{-0.45}$	$2.11^{+0.49}_{-0.52}$	$2.06^{+0.49}_{-0.45}$	$2.11^{+0.48}_{-0.52}$
$1.5n_{\rm sat}$	$6.06^{+1.85}_{-2.04}$	$6.25^{+1.90}_{-2.26}$	$6.08^{+1.83}_{-2.04}$	$6.25^{+1.89}_{-2.23}$
$2.0n_{\rm sat}$	$19.47^{+33.63}_{-11.67}$	$19.07^{+15.27}_{-10.53}$	$19.35^{+33.66}_{-10.71}$	$19.05^{+15.33}_{-10.27}$
$2.5n_{\rm sat}$	$47.78^{+75.96}_{-32.96}$	$45.43^{+40.41}_{-19.11}$	$47.59^{+79.68}_{-27.46}$	$45.57^{+40.87}_{-18.89}$
$1.0M_\odot$	$11.89^{+0.79}_{-0.98}$	$11.88^{+0.57}_{-0.76}$	$11.89^{+0.79}_{-0.98}$	$11.88^{+0.56}_{-0.78}$
$1.4M_\odot$	$12.06^{+1.13}_{-1.18}$	$12.01^{+0.78}_{-0.77}$	$12.06^{+1.12}_{-1.19}$	$12.01^{+0.78}_{-0.77}$
$1.6M_\odot$	$12.11^{+1.33}_{-1.33}$	$12.03^{+0.98}_{-0.75}$	$12.11^{+1.32}_{-1.34}$	$12.03^{+0.92}_{-0.80}$
$2.0M_\odot$	$12.19^{+1.71}_{-1.59}$	$11.91^{+1.24}_{-1.11}$	$12.18^{+1.70}_{-1.61}$	$11.91^{+1.17}_{-1.15}$

5 Comparison of the 95% credible interval for the pressure $[\,\mathrm{MeV\,fm^{-3}}]$ and radius [km] of neutron stars when including only HIC experiments and for combined HIC and astrophysics results with and without the inclusion of the constraint from Danielewicz *et al.*[27]. By comparing the HIC-only results, we conclude that the constraint from Danielewicz *et al.*[27] has a small impact on our study.
